# Several Follicular Regulatory T Cell Subsets With Distinct Phenotype and Function Emerge During Germinal Center Reactions

**DOI:** 10.3389/fimmu.2018.01792

**Published:** 2018-08-13

**Authors:** Nicolas Fazilleau, Meryem Aloulou

**Affiliations:** ^1^Centre de Physiopathologie de Toulouse Purpan, Toulouse, France; ^2^INSERM U1043, Toulouse, France; ^3^CNRS UMR5282, Toulouse, France; ^4^Université Toulouse III Paul-Sabatier, Toulouse, France

**Keywords:** T follicular regulatory cells, germinal centers, heterogeneity, subsets, T follicular helper cells

## Abstract

An efficient B cell immunity requires a dynamic equilibrium between positive and negative signals. In germinal centers (GCs), T follicular helper cells are supposed to be the positive regulator while T follicular regulatory (Tfr) cells were assigned to be the negative regulators. Indeed, Tfr cells are considered as a homogenous cell population dedicated to dampen the GC extent. Moreover, Tfr cells prevent autoimmunity since their dysregulation leads to production of self-reactive antibodies (Ab). However, a growing corpus of evidence has revealed additional and unexpected functions for Tfr cells in the regulation of B cell responses. This review provides an overview of the Tfr cell contribution and presents Tfr cell proprieties in the context of vaccination.

## Introduction

One of the key roles of humoral response is to clear pathogens and to prevent future pathogen assaults through the induction of immune memory. This long-term protection is largely mediated by the generation of high-affinity and neutralizing antibodies (Ab) bearing the suitable isotype for pathogen clearance. However, the increase of B cell receptors affinity in germinal centers (GCs) is mediated by somatic hypermutation, which is a random process mediated by the enzyme activation-induced cytidine deaminase. Therefore, affinity maturation requires tight regulation processes of mutagenesis and B cell selection that are essential in GCs to guarantee proper surveillance and to avoid sustained activation that could lead to autoimmunity or inflammatory diseases. GCs represent critical sites within secondary lymphoid organ (SLO) in which B cell responses are amplified and refined in specificity and isotype, leading to the generation of high-affinity memory B cells and long-lived plasma cells (PCs). The cellular mechanisms that control positive selection of GC B cells have been mostly elucidated. Entanglement of B cells with T follicular helper (Tfh) cells is at the center of this selection process (1). Indeed, Tfh cells seed primordial GCs and provide positive help to the selected GC B cells bearing high-affinity Ab. Several signals provided by Tfh cells lead to B cell maturation (2). Tfh cells bear T cell receptor (TCR) with high affinity for the immunizing antigen (Ag), which lead to stable interaction with GC B cells bearing abundant complexes of foreign peptide–MHC complexes (pMHCII) (3–5). T–B cell entanglement rarely lasts more than 10–15 min but triggers the activation of signaling cascades in B cells and cytokine secretion by Tfh cells that promote survival, proliferation, mutagenesis, and terminal differentiation of B cells into PCs and memory B cells (1, 2). This interaction also provides additional helper signals from Tfh cells through co-stimulatory molecules (2). Furthermore, the output of GCs varies depending on the nature of the Ag and the type of inflammation. Several immunoglobulin isotypes exist, which classes are directed by the type of pathogen and the inflammatory context. In addition, specialized subsets of Foxp3^+^ regulatory T cells (Treg), the T follicular regulatory (Tfr) T cells, were also recently found in GCs of mice (6–8) and human (9), where they play an immunosuppressive function. Tfr cells express cytotoxic T-lymphocyte antigen 4 (CTLA-4), glucocorticoid-induced tumor necrosis factor receptor (GITR), inducible T-cell co-stimulator (ICOS) and produce IL-10, a phenotype that is the characteristic of activated Treg. Until now, the regulation processes of B cell selection have only been assigned to Tfh cells. However, a recent study has shown the ability of Tfr cells to promote proliferation of GC B cells through IL-10 provision (10). In addition, conflicting results were obtained regarding the role of Tfr cells in controlling affinity maturation of B cells in response to a foreign Ag (see Table 1) (6, 7). All these observations highlight the multifaceted role of Tfr cells and the requirement for further studies to unravel their exact functional proprieties.

**Table 1:** Impact of Tfr cell abnormalities on B cell maturation.

Tfr cell abnormality	Ag model	Impact on B cell response	Reference
Tfr deficiency (Bcl6^−/−^ Treg and WT naïve Th cells transferred into Tcr^−/−^ mice)	NP-KLH in CFA	Increase high-affinity IgG1, IgG2a, and IgG2b Ab	(6)

Tfr deficiency (bone marrow chimeras SAP^−/−^:Foxp3^DTR^)	NP-KLH in SAS	Reduce high-affinity Ab after boost	(7)

Tfr deficiency (CXCR5^−/−^ Treg and OVA specific Th cells transferred into Tcr^−/−^ mice)	OVA in Alum	Increase Ag-specific IgM and IgG2b Ab	(8)

Treg cells-lacked IL-10 production (Foxp3cre × IL-10^fl/fl^)	LCMV Armstrong infection	Reduce LCMV-specific Abs bearing IgG1 and IgG2a isotype	(10)

PD-1 deficiency (WT Tfh and Pdcd1^−/−^ Tfr cells transfer into CD28^−/−^)	NP-OVA in CFA	Reduction of Ag-specific Ab	(61)

CTLA-4 deficiency (Foxp3cre ERT2 × Ctla-4^fl/fl^ Tfh and Tfr cells transfer into CD28^−/−^ and tamoxifen administration)	NP-OVA in CFA	Increase Ag-specific IgG	(76)

Tfr deficiency (Bcl6^−/−^ CD25^+^ Th cells and WT naïve Th cells transferred into CD3^−/−^ mice)		Reduce IgA titers	(83)
		Reduce IgA maturation and microbiota diversification	

Tfr deficiency (Foxp3cre × Bcl6^fl/fl^)	– HIV vaccine model – NP-KLH in Alum	Reduce Ab avidity	(85)
		Reduce Ag-specific IgG	
		Increase Ag-specific IgA	

*SAS, Sigma Adjuvant System; NP-KLH, 4-hydroxy-3-nitrophenyl-Keyhole Limpet Hemocyanin; OVA, Ovalbumin, CFA, Complement Freund’s Adjuvant; SAP, Signaling lymphocytic activation molecules (SLAM)-associated protein; LCMV, Lymphocytic Choriomeningitis; Tfr, T follicular regulatory; CTLA-4, cytotoxic T-lymphocyte antigen 4; Bcl-6, B-cell lymphoma 6; PD-1, protein cell death 1; Treg, regulatory T cells; Tfh, T follicular helper*.

## Tfh Cells, the Positive Regulators of GCs

After initial priming with Ag-experienced dendritic cells (DC), Ag-specific Th cells are clonally selected, expand drastically and, depending on the cytokine milieu and co-stimulatory signals, develop into different lineages of effector T cells such as Th1, Th2, and Th17 or into a lineage of suppressor cells, the periphery Tregs (pTreg). While it has long been thought that Th2 cells were the specific helper of B cells, it is now clear that the guidance of B cell responses is under the control of specific cognate regulators, namely the Tfh cells (2, 11, 12). Early analyses revealed a specific transcription profile for Tfh cells, which was distinct from those of Th1-, Th2-, and Th17-polarized cells and identified a suite of key surface markers that discrimate Tfh cells from the other effector Th cell lineages (13). Several studies have shown that the repressor B-cell lymphoma 6 (Bcl-6) drives the genetic program imprinted in Tfh cells (14–17). Bcl-6 expression is dependent on different transcription factors that regulate the key targets of human and mouse Tfh cell formation such as achaete-scute homolog 2 (18), signal transducer and activator of transcription 3 (Stat3) (19), IFN regulatory factor 4 (20, 21), and c-Maf (22). Phenotypically mouse Tfh cells are CCR7^lo^ CXCR5^hi^ (23, 24) and CD45RA^−^ CXCR5^+^ cells are also greatly enriched in human tonsils and located in the B follicle areas of these inflamed tissues (25). In addition to these surface molecules that define their strategic anatomical position, Tfh cells highly express molecules essential for their B cell helper function. These molecules include protein cell death 1 (PD-1), B and T lymphocyte attenuator, CD40L, ICOS, SAP, and the production of IL-21 and IL-4 (2). ICOS engagement is important for IL-21 production by Tfh cells (26). However, additional cytokines such as IFN-γ, IL-13, IL-5, and IL-17 can be produced by the polarized Tfh cells depending on the inflammatory context (27). These cytokines promote B cell isotype switch to different pathogen challenges (28). Interestingly, under conditions of intense polarization mouse GC Tfh cells can express Th1-, Th2-, or Th17-differentiation program such as T-bet (T-box expressed in T cells) and IFN-γ, IL-5 and IL-13 or Rorγt (retinoic acid receptor-related orphan nuclear receptor gamma t) and IL-17, respectively (29–32). In human, few GC Tfh cells display polarized phenotype according to the production of non-Tfh cell cytokines in SLO (33, 34). However, circulating Tfh cells express chemokine receptors corresponding to the polarized non-Tfh cell subsets Th1, Th2, and Th17 cells (26, 27). Indeed, circulating human Tfh cell compartment can be stratified into three distinct polarized subsets based on their expression of chemokine receptors: CXCR3^+^CCR6^−^ Tfh1-, CXCR3^−^CCR6^−^ Tfh2-, and CXCR3^−^CCR6^+^ Tfh17-like cells (26, 27). Overall, depending on the inflammatory context, different Tfh cell profiles are observed reflecting the heterogeneity of the Tfh cell compartment either in mice or human.

## Tfr Cells, other GC Regulators

### Tfr Cell Development and Antigen-Specificity

Regulatory T cells form the main population of immunosuppressive T cells that plays a pivotal role in maintaining immune self-tolerance and homeostasis by suppressing aberrant or excessive immune responses deleterious to the host (35). Treg use the appropriate homing receptors to control their migration to the site of inflammation and use relevant immunosuppressive mechanisms. In order to repress Th1-, Th2-, and Th17-mediated immune responses, Treg have been shown to co-opt selective aspects of the differentiation programs required for these Th cell lineages (36–38). In the context of B cell responses, Treg were also shown to have the capacity to express Bcl-6 and the chemokine receptor CXCR5, which allows migrating into CXCL13 rich areas to control the GC response (39, 40). These cells were coined as Tfr cells. Using Treg depletion and adoptive transfer, it was initially proposed that Tfr cells derive only from thymus-derived Treg (tTreg) (6–8). However, we recently demonstrated that Tfr cells can also derive from naïve Th cells, a process that required PD-1 ligand 1 (PD-L1) signaling (41). Such differentiation occurs if the adjuvant used is one that supports naive Th cell conversion to pTreg such as incomplete Freund’s adjuvant (IFA) (41). Tfh cells regulate GC B cells by interacting with these cells in a cognate fashion, demonstrating that Tfh cells are specific for the Ag against which the ongoing immune response is mounted. Regarding the Ag-specificity of Tfr cells, our studies also demonstrated that a fraction of these cells could be specific for the immunizing Ag, irrespective of whether it is a self or a foreign Ag (41). Our observations were recently challenged by the fact that it was found that the TCR repertoires of Tfh cells and Tfr cells were shown to be largely distant (42). Moreover, it was also shown the TCR repertoire of Tfr cells was the closest to the one of Treg (42). Finally, only few ovalbumin (OVA)-specific Tfr cells could be detected using pMHCII tetramers in the draining lymph nodes 11 days post-immunization with OVA/IFA (42). Overall, the authors concluded that Tfr cells and Tfh cells do not share the same Ag-specificity and that Tfr cells originate only from tTreg and bear auto-reactive TCRs to suppress autoimmunity (43). We suggest that the inflammatory environment and the nature of the Ag actually both dictate whether Tfr cells can arise from pTreg, which ultimately influence the Tfh and Tfr TCR repertoire overlap and the proportion of Tfr cells sharing the same Ag-specificity with Tfh cells. Indeed, tTreg are thought to be largely auto-reactive (44). Nonetheless, tTreg specific for foreign epitopes have also been described in the naïve population (45), as well as during infection (46, 47). More precisely, the thymic origin of Treg specific for a non-self Ag have been investigated in naïve mice by using tetramer-based enrichment method (45). This corroborates previous studies showing that the TCR repertoire of the Treg population is as diverse as the one of conventional Th cells (48–50), which explains the capacity of tTreg TCRs to possibly cross-react with pMHCII complexes presenting foreign Ag. In the context of *Leishmania major* or *Influenza* infection, Treg were shown to strongly proliferate suggesting that the TCRs of these cells recognized microbe derive Ag (46, 51, 52). Moreover, in the context of *Mycobacterium tuberculosis* (Mtb) infection (46), it was clearly demonstrated using pMHCII tetramers that Mtb-specific Treg expanded from the pre-existing pool of tTreg and displayed distinct TCR repertoire as compared to the one of the Th cells while they were sharing the same Ag-specificity to Mtb. Elegantly, the authors also showed that a recombinant strain of *Listeria monocytogenes* (Lm) expressing the Mtb immunodominant ESAT6 epitope induced the proliferation of ESAT6-specific conventional Th cells but not of ESAT6-specific Treg, suggesting that the inflammatory milieu of Mtb, but not of Lm, promotes the expansion of Ag-specific tTreg (46). Finally, in the context of self-reactivity, it was shown that myelin oligodendrocyte glycoprotein (46)-specific tTreg expressed TCR of higher avidity than conventional Th cells, suggesting that, despite the same Ag-specificity, their TCR repertoires were different (53). Overall, these data suggest that the control of humoral responses may be defined by distinct Tfr cell subsets, either specific or not for the immunizing Ag, and ultimately GC B cells could be regulated by Tfr cells through non-cognate and cognate interactions.

### Tfr Cell Differentiation

The transcriptional program essential for Tfr cells formation was recently described. Most of the genes are common with the Tfh cell program such as Bcl-6, Stat3, and Tcf-1 (54), but specific genes to the Tfr cell lineage are also found such as Nfat2 that initiates CXCR5 expression on Treg (55). Mechanistically, mTOR kinase complexes 1 and 2 (mTOR1 and mTOR2) are involved in Tfh and Tfr cell differentiation. More precisely, both mTOR1 and mTOR2 are essential for Tfh cell formation by linking immune signals to anabolic metabolism and transcriptional activity (56, 57). In addition, mTOR1, but not mTOR2, mediates Tfr cell differentiation by activating the Stat3/Tcf-1/Bcl-6 axis (54). Similar to Tfh cells, initial Tfr cell formation requires engagement of several surface molecules such as CD28, receptors associated to SAP and ICOS that all lead to sustained interaction with Ag-presenting cells (APC) such as DC or B cells. T-cell priming through CD28 is the first signal required for Tfh and Tfr cell development (7, 58), while the adaptor protein SAP enables the formation of stable interaction with B cells essential for Tfh and Tfr cell differentiation (7, 59). ICOS leads to sustained Bcl-6 expression by Tfh and Tfr cells through activation of p85α regulatory subunit of the PI3-kinase and intracellular ostepontin (60).

In order to prevent full suppression of the GC reaction, a panel of negative regulators was also shown to counterbalance the positive signals that lead to Tfr cell differentiation. PD-1 limits both the differentiation and suppressive function of Tfr cells after their binding to PD-L1 but not to PD-L2 (61). Unlike Tfr cells, PD-1 deficiency has no effect on GC Tfh cell number, while frequency of circulating Tfr and Tfh cells are greater in the blood, suggesting that both Tfh and Tfr cells are repressed by PD-1 signaling (61). The helix-loop-helix proteins Id2 and Id3 are other suppressive mechanisms of Tfr cell development. Initial TCR engagement of Treg decreases the abundance of Id2 and Id3, which both contribute to the activation of the Tfr cell specific transcription program (62). Interestingly, in contrast to fully differentiated Tfh cells, Tfr cells co-express the antagonistic regulators B-lymphocyte-induced maturation protein 1 (Blimp1) and Bcl-6. Such co-expression could limit the number of Tfr cell as highlighted by Blimp1 deficiency that does not alter Tfh cell development but causes an increase of the Tfr cell proportion (7). This observation is in contrast with published data showing that Blimp1 directly limits global follicular T cell formation (14, 63), however, its impact on Tfh and Tfr cells separately has not been explored.

Recent studies have described the influence of cytokines on Tfr cell differentiation and maintenance. IL-21/IL21-receptor interaction limits the proliferation of Tfr cells (64). In this study, the authors demonstrated that IL-21 restricts Tfr proliferation by limiting CD25 expression and responsiveness to IL-2, through a Bcl-6-dependent mechanism (64). Furthermore, IL21R-deficiency in mice and human increases Treg and Tfr cell numbers (64). In another series of study, Botta et al. showed that IL-2 prevents Tfr cell development through Blimp1 mechanisms (65) and that Tfr cells express low level of CD25. However, Tfr cells are not completely irresponsiveness to IL-2. Tfr cells express high amount of intermediate affinity IL2-R, CD122 (65), which could promote the IL-2–STAT5 axis important for maintaining Foxp3 expression. Consequently, both Tfh and Tfr cell developments are restrained by IL-2 cytokine. In the context of *Influenza* infection, the presence of high amount of IL-2 in the early phase leads to complete abrogation of Tfr cell formation while Tfh cells are maintained (65). In this context, it was shown that Tfr cells appear lately once the immune response resolves, which may contribute to prevent the expansion of self-reactive B cells. By contrast, in the context of protein vaccination, the Tfr cells follow the same formation and resolution kinetics than Tfh cells (7, 41), which could result from a different IL-2 profile. Therefore, the dynamic of Tfr cell development does not carry a single form but is closely related to the inflammatory context.

T follicular regulatory cells, like Tfh cells, can exit the draining SLO and join the circulation in both mice (66) and human (67, 68). The circulating Tfr cells (cTfr) have been shown to expand after protein immunization or viral infection. cTfr development requires priming by DC in draining SLO, these cells leave the SLO before GC formation (66). In human, cTfr cells can also be generated before T–B interaction, as they are maintained in B-cell-deficient patients (43). cTfr cells share proprieties of memory cells and persist for long lasting period *in vivo* and present distinct proprieties as compared to GC-Tfr cells. Indeed, cTfr cells express less ICOS and present less suppressive functions (66). Upon second immunization, cTfr cells home to GC and suppress Tfh and B cell activation irrespective of their Ag specificity. Therefore, the limited suppressive capacity of cTfr cells may contribute to improve the performance of memory Tfh cell response thereby enabling productive recall Ab responses. Even if the cTfr cells exit the SLO before GC reaction (66, 67), many studies have used the ratio of circulating Tfr and Tfh cells as an indicator of the ongoing GC reaction during autoimmune diseases such as systemic lupus erythematosus (68, 69), multiple sclerosis (70), rheumatoid arthritis (71), and Sjögren’s syndrome (72). Other studies also explored the cTfr cell frequency in response to foreign Ag after vaccination or infection. During chronic hepatitis B and chronic hepatitis C, an increase number of cTfr cells in patients was associated with poor virus eradication and liver injury (73). By contrast, after flu vaccination, cTfr cell frequencies increased and correlated with enhanced anti-flu Ab responses (70). Due to the limited access to SLO organs from humans, the circulating follicular T cells represent an ideal indicator of B cell responses even if the ratio of cTfr/cTfh cells corresponds to a biased marker of GC events. Therefore, a better characterization of cTfr cell development and function during physiological and pathological contexts is required in order to perform an appropriate assumption of the GC reaction.

## Multifaceted Tfr Cell Function during GC B Cell Selection

### Tfr Cells Are Negative Regulators

T follicular regulatory cells have the surface profile of Tfh cells (CXCR5^hi^ PD-1^hi^ ICOS^+^) and localize in the GC, but they also express Foxp3 and exhibit a CTLA-4^hi^, GITR^hi^, ICOS^hi^, and IL-10^hi^ phenotype that is the characteristic of activated Treg. CTLA-4 and PD-1 are known to enhance the suppressive activity of Treg (74, 75). Tfr cells express these molecules uniformly, however, CTLA-4 and PD-1 display distinct Tfr cell functions. CTLA-4 deficiency leads to a decrease production of Ag-specific Ab (76) through mechanisms that either alter (77) or not (76) the co-stimulatory signals provided by GC-B cells. By contrast, PD-1 deficiency leads to an increase of the suppressive activity of Tfr cells (61). Importantly, many molecules involved in Tfr cell differentiation are also important in Tfr cell function. As an example, alteration of the mTOR1 signaling pathway in differentiated Tfr cells leads to decreased expression of CTLA-4, ICOS, and PD-1, which consequently leads to a decrease of Tfr cell suppressive activity (54). Tfr cells express many other Treg cell inhibitory molecules such as GITR, granzym A, and CD103, however, whether these molecules participate to the regulation of Ab production by Tfr cells still remain unknown.

The suppressive function of Tfr cell leads to durable and persistent inhibition of B cells through epigenetic modifications (78). B cells suppressed by Tfr cells decrease expression of genes involved in metabolic pathways and class switch recombination. Tfr cells also suppress genes involved in Tfh cell effector functions such as IL-4 and IL-21. Interestingly, IL-21 produced by Tfh cells overcomes the Tfr-suppressive function by stimulating B cell metabolism and function. IL-21 might alter Tfr cell metabolism and thereby reduces suppressive activity, as has been observed in Treg cells (79, 80). Therefore, IL-21 secretion might be a key factor in balancing the B cell and Tfr cell activity.

While Tfr cells arise from Treg and use many of the Treg attributes to regulate GCs, these cells have also their proper mechanisms. Unexpectedly, it was shown very recently that Tfr cells do not express CD25 (81). IL-2 promotes Treg proliferation (82). By contrast, it was demonstrated that IL-2 inhibits Tfr cell formation after *Influenza* infection (65). Moreover, while the Treg/Th cell balance is regulated by the IL-2 axis, the Tfr/Tfh cell balance is regulated by the IL-1 axis (81). Tfh cells express the agonist receptor IL-1R1 that promotes Tfh cell activation in response to IL-1. By contrast, Tfr cells express the IL-1 decoy receptor IL-1R2 and the IL-1 receptor antagonist IL-1Ra. Consequently, Tfr cells limit Tfh cell activation by limiting IL-1 availability within the GC (81). Thus, the dialog between effector and Treg use appropriate mechanisms depending on its location: IL-2 axis by Treg cells in the T cell zone vs IL-1 axis by Tfr cells in the B cell follicle.

### Other Functions of Tfr Cells

Beside the regulatory phenotype of Tfr cells, suppressive machinery during GC B cell selection can provide unexpected positive functions to Tfr cells (see Table 1). Several studies have demonstrated a role for Tfr cells in the control of the affinity maturation of B cells in response to foreign Ag (6–8) or within the gut where IgA production allows microbiota diversification (83). However, conflicting results were obtained regarding how Tfr cells control B cell maturation. The most probable reason for this divergence is related to the different experimental systems used to deplete Tfr cell population. One of these strategies was the use of mixed bone marrow chimera of SAP-deficient mice and DEREG mouse model, in which diphtheria toxin (DT) receptor expression is under the control of the Foxp3 promoter. Treatment of these chimeras with DT leads to selective depletion of Tfr cells (7). As SAP is involved during APC/T and T/T cell interactions (84), its absence in Treg may contribute to the modulation of immune responses and expansion of Tfh cells. Other strategies consisted in the co-transfer of naive CD4^+^ T cells with either Bcl6- or CXCR5-deficient Treg into T cell-deficient mice (6, 8, 83). In this context, the lymphopenia of T cell-deficient mice can lead to T cell expansion and therefore may mask the function of Tfr cells in regulating the Tfh cells amplitude. A recent study used a novel mouse model by crossing the Foxp3cre mouse strain with Bcl6floxed mice, which rendered possible the complete depletion of the Tfr cell compartment and left intact the Treg pool (85). Unexpectedly, following immunization with non-self Ag in these animals, Tfr cell deficiency showed no impact on the Tfh and GC B cell magnitude. By contrast, Tfr cell deficiency led to a decreased avidity of Ag-specific Ab, a decrease of Ag-specific IgG and an increase of IgA titers. In the context of autoimmunity using the pristane-induced lupus model in these mice, Tfr cell deficiency led to an increase of anti-dsDNA IgA titers while similar titers of anti-dsDNA IgM and IgG were observed. Finally, absence of Tfr in gut-associated lymphoid tissues led to a decrease of the helper properties of Tfh cells, notably by reducing IL-21 production (83). Overall, these studies demonstrated that Tfr cells do not only control GC reaction magnitude but can also control the downstream events resulting in class switching and maturation and, ultimately, optimize GC responses by increasing Ab avidity, promoting IgG class switch, repressing abnormal IgA responses, and preventing the production of self-specific Ab. Efficient GC reaction relies on the fine regulation of the processes of somatic hypermutation and class switch recombination during B cell clone proliferation within the dark zone (DZ) and of GC B cell selection in the light zone. These sequential events depend on increasing the surface of entanglement between GC Tfh and GC B cells through co-stimulatory molecules and TCR engagement, limiting the number of GC Tfh cells in order to increase the threshold for GC B cell selection and controlling the cytokine production by GC Tfh cells to orientate the class switching. Therefore, the multifaceted function of Tfr cells may reflect the function of different subsets of Tfr cells (see Table 2). This heterogeneity in the Tfr cell compartment could also explain the divergence in the results obtained when studying the impact of Tfr cell deficiency.

**Table 2:** List of the different T follicular regulatory (Tfr) cell subsets and their putative functions.

Tfr cell subsets	Putative function	Reference
Tfr cells derived from thymic regulatory T cells	Maintain tolerance and restrain magnitude of GC B cells	(6–8)

Tfr derived from naïve Th cells	Mostly specific for the immunizing Ag allowing the control of Ag-specific B cell response through cognate interaction	(41)

IL10-producing Tfr cells	Promote plasmablast differentiation and GC development	(10)

PD-1^+^ Tfr cells in mouse and PD-1^lo^ in human	Abundant population that displays less suppressive activity preventing excessive suppression of GC reaction	(61, 86)

PD-1^+^ Tfr cells in human	Display greater suppressive activity (TGFβ^hi^, IL-10^hi^, CTLA-4^hi^)	(86)

Circulating Tfr cells	Display less suppressive capacity and home to GC upon recall contributing to faster memory T follicular helper cell response	(66)

One attribute of Tfr cell subdivision could depend on the Tfr cell ontogeny. The Tfr cells deriving from pTreg are mostly specific for the immunizing Ag while only a fraction of Tfr deriving from tTreg shares its Ag specificity with Tfh cells (41). Therefore, the Tfr cells can be stratified into two distinct populations according to their Ag-specificity. The non-Ag-specific Tfr cells could restrict the outgrowth of non-Ag-specific GC B cells while the Ag-specific Tfr cells could control, in a cognate fashion, the extent of the Ag-specific GC B cells. Moreover, the Ag-specific Tfr cells could play a crucial role in the selection mechanisms of high-affinity B cell clones. The competition for T cell help promotes affinity maturation of B cells. It is conceivable that Ag-specific Tfr, as they could compete with GC Tfh cells to interact with pMHCII at the surface of GC B cells, could limit Tfh/B cell interaction and consequently could lead to an increase affinity maturation of B cells. Interestingly, human Tfr cells also display phenotypic diversity, according to PD-1 expressing (86). Indeed, PD-1^+^ and PD-1^low^ Tfr cells were characterized in human mesenteric lymph nodes with high abundance of PD-1^low^ Tfr cells. Sage et al. showed that PD-1-deficient Tfr cells were more suppressive than PD-1-sufficient Tfr cells (61). However, human PD-1^+^ and PD-1^low^ Tfr cells suppress Ab responses *in vitro* with the same efficacy (86). Therefore, PD-1 expression on Tfr cells could have distinct function in human and mouse. While PD-1 expression decreases the suppressive activity of mouse Tfr cell, PD-1 expression on human Tfr cells could modulate the Abs responses *in vivo*. Indeed, human PD-1^+^ Tfr cells express high levels of CD38, CTLA-4, and GARP, a protein critical for the surface expression of latent TGFβ in activated human Treg cells (87), as compared to PD-1^low^ Tfr cells (86).

**Figure 1:**
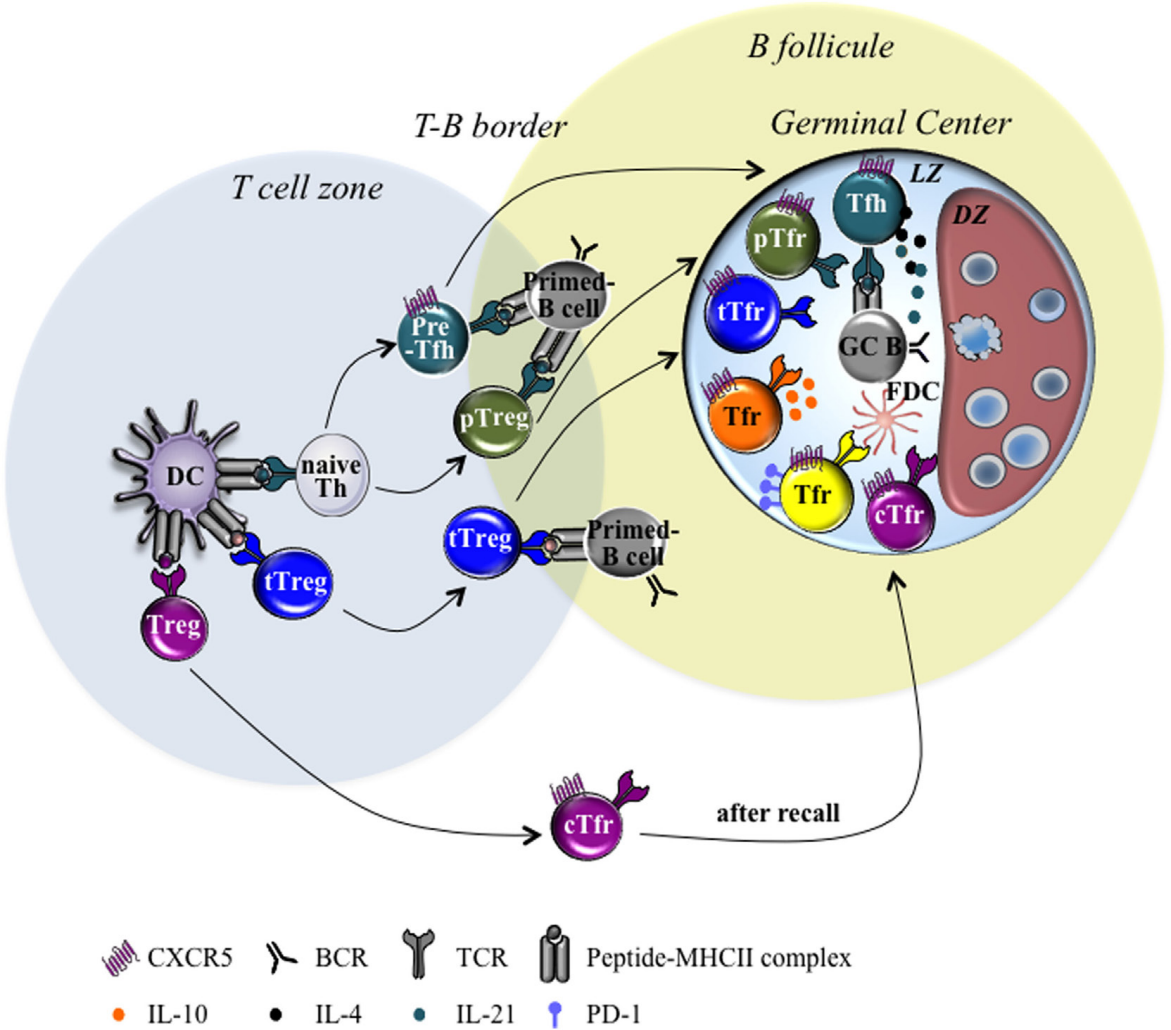
Heterogeneity of T follicular regulatory (Tfr) cells. Schematic diagrams illustrating the different Tfr cell subsets found within germinal center (GC) in second lymphoid organs draining the site of infection or immunization. Tfr cell development relies on sequential events. First, differentiation of naive Th cells into pre-Tfh cells or periphery Tregs (pTreg) cells depends on the signals provided by dendritic cells (DC) during TCR/pMHCII cognate interaction. Then, thymus-derived Treg (tTreg) and pTreg differentiate into Tfr cells in response to the signals provided by DC and/or B cells. Consequently, Tfr cells can be subdivided into different cell subsets according to several features: (i) origin: thymic-derived Tfr (tTfr) cells, Tfr derived from pTreg (pTfr) and circulating Tfr (cTfr) cells; (ii) Ag specificity: Tfr cells sharing or not the same Ag-specificity with Tfh cells; (iii) produced cytokine: IL-10 secreting and IL-10 non-secreting Tfr cells; (iv) surface molecule: PD-l^hi^ and PD-1^low^ Tfr cells. Abbreviations: TCR, T cell receptor; pMHCII, peptide–MHC complexes; FDC, follicular dendritic cell; DZ, dark zone; LZ, light zone; PD-1, protein cell death 1; Tfh, T follicular helper.

T follicular regulatory cell subdivision could also rely on the cytokines produced by these cells, such as IL-10 (10). Indeed, a recent study has shown the ability of Tfr cells not to suppress but to promote GC response through provision of IL-10, an anti-inflammatory cytokine (10). More precisely, specific ablation of IL-10 in Tfr cells only led to a decrease of the GC response. IL-10 was shown to act specifically on B cells and to promote DZ phenotype through the induction of the nuclear factor FOXO1 and to potentially enhance their affinity maturation (10). IL-10-secreting and non-secreting Tfr cells could represent two distinct populations. Even if the two populations express CTLA-4, they could dialog with distinct cell targets and could have different functional outcomes. Finally, as stated already, cTfr cells emerge before GC reaction and are able to home to GC during secondary response to suppress GC Tfh and B cell activation (66, 67). Therefore, GC reaction that contains cTfr-derived Tfr cells could restrain the generation of new-formed GC-Tfr cells and could display less suppressive activity, which could contribute to improve the performance of memory B cell response. Overall, the Tfr cell population that enters the GC reaction can rely on different features (origin, Ag specificity, cytokine produced, expression of surface molecules, etc.) that could impact the Tfr cell function and, consequently, the quality of the B cell response (see **Figure 1**).

## Conclusion

Humoral responses have a pivotal role in the development of protective immune responses. Follicular T cells form the main T cell population controlling GC reaction. Over time, follicular T cells have been broadly characterized and stratified into different specialized subsets providing B cell helper function. More recently, Tfr cells have been identified among the follicular T cells. While, these Tfr cells have been initially described as negative regulators, many new evidence demonstrate that Tfr cells do not act as passive inhibitor but can integrate the environmental cues and achieve adapted program to regulate the corresponding type of immune response within the GC. These proprieties are mandatory to get a correct cell communication within the GC and to guaranty relevant B cell maturation. Therefore, better characterization of the properties of Tfr cell subsets in regulating GC reaction is mandatory and could allow defining new vaccination strategies through selective modulation of particular Tfr cell subsets.

## Author Contributions

NF and MA conceived and wrote the manuscript.

## Conflict of Interest Statement

The authors declare that the research was conducted in the absence of any commercial or financial relationships that could be construed as a potential conflict of interest.
